# Laparoscopic surgical management of a mature presacral teratoma: a case report

**DOI:** 10.1186/s40792-019-0702-x

**Published:** 2019-09-18

**Authors:** Liming Wang, Yasumitsu Hirano, Toshimasa Ishii, Hiroka Kondo, Kiyoka Hara, Shintaro Ishikawa, Takuhisa Okada, Nao Obara, Shigeki Yamaguchi

**Affiliations:** Department of Gastroenterological Surgery, Saitama Medical University International Medical Center, 1397-1 Yamane, Hidaka-shi, Saitama, 350-1298 Japan

**Keywords:** Retrorectal, Mature teratoma, Laparoscopic

## Abstract

**Background:**

Mature presacral (retrorectal) teratoma is very rare. We report a case in which a mature retrorectal teratoma in an adult was successfully treated with laparoscopic surgery.

**Case presentation:**

A 44-year-old woman was diagnosed with a presacral tumor during a physical examination. Endoscopic ultrasonography (EUS) revealed a multilocular cystic lesion; the lesion was on the left side of the posterior rectum and measured approximately 30 mm in diameter on both contrast-enhanced pelvic computed tomography (CT) and magnetic resonance imaging (MRI). The presumptive diagnosis was tailgut cyst. However, the histopathological diagnosis after laparoscopic resection was mature teratoma. It is still difficult to preoperatively diagnose mature retrorectal teratomas.

**Conclusions:**

Laparoscopic resection of mature retrorectal teratomas is a feasible and promising method that is less invasive and can be adapted without extending the skin incision.

## Background

Teratomas are composed of mesodermal, endodermal, and ectodermal tissue. The cystic components of teratomas mainly contain sebum and hair [1, 2]. Teratomas can also have mature elements composed of normal cells that have differentiated into cells of one of the three embryonic germ layers. The ovary is the most commonly reported site of primary teratomas in adult women, followed by the retroperitoneum and sacral cauda. Most are asymptomatic, do not involve any specific complaints [3], and so are found incidentally during routine physical examinations. However, any of the components of a teratoma can undergo malignant transformation, with the reported incidence of malignant change ranging from 1 to 12% [3–5]. Mature teratoma is a surgical diagnosis, and surgery is the primary treatment [6]. We present a case of successful laparoscopic resection of a mature retrorectal teratoma without malignant transformation.

## Case presentation

**Fig. 1:**
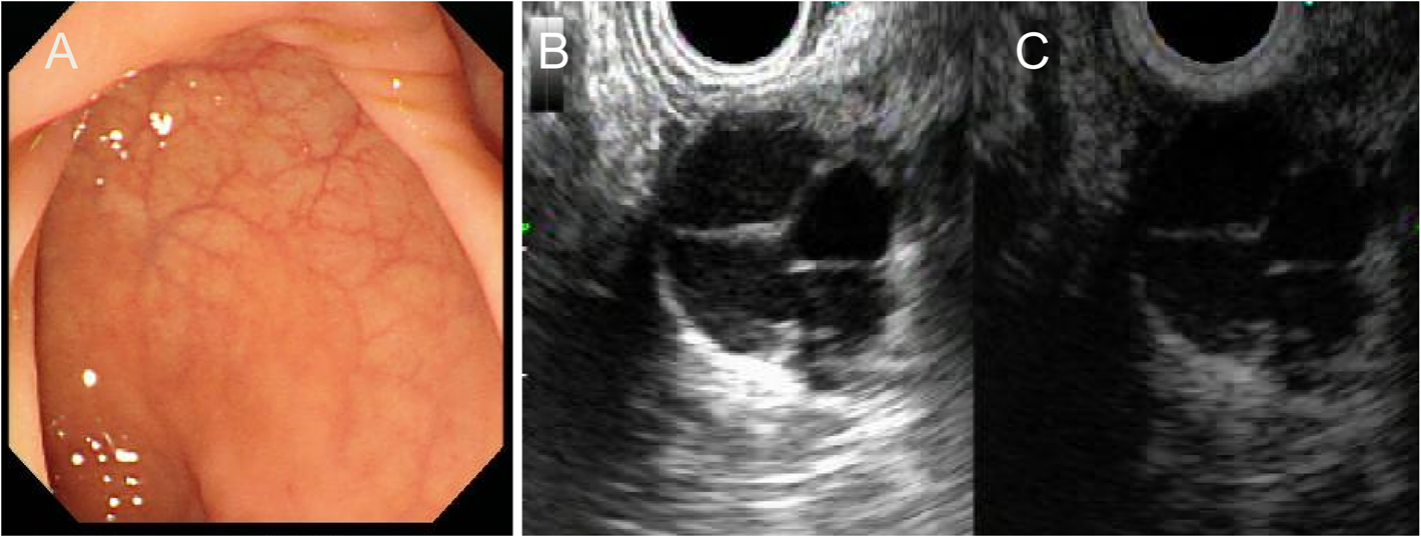
Colonoscopy and endoscopic ultrasound. **a** No rectal submucosal tumor is seen on colonoscopy. **b** The endoscopic ultrasonography (EUS) shows a hypo- and anechoic multilocular mass measuring 20 mm in diameter with clear boundaries. **c** Only the cyst wall is enhanced on contrast-enhanced ultrasonography (CE-US)

A 44-year-old woman presented with a submucosal rectal mass found incidentally during a physical examination. On digital rectal examination, the mass was soft with a smooth surface and was located on the left posterior wall of the rectum. The results of routine laboratory tests were all within the normal range. The patient underwent colonoscopy with endoscopic ultrasonography (EUS), but EUS-guided needle biopsy was deferred to avoid the risk of tumor seeding or infection in the pelvic cavity. The ultrasound showed a multilocular hypo- and anechoic 20-mm mass on the posterior wall of the rectum, which exhibited clear boundaries. Some of the tissue had calcified, and there was no internal blood flow. Only the cyst wall was enhanced by contrast during contrast-enhanced ultrasonography (CE-US) (Fig. 1).

**Fig. 2:**
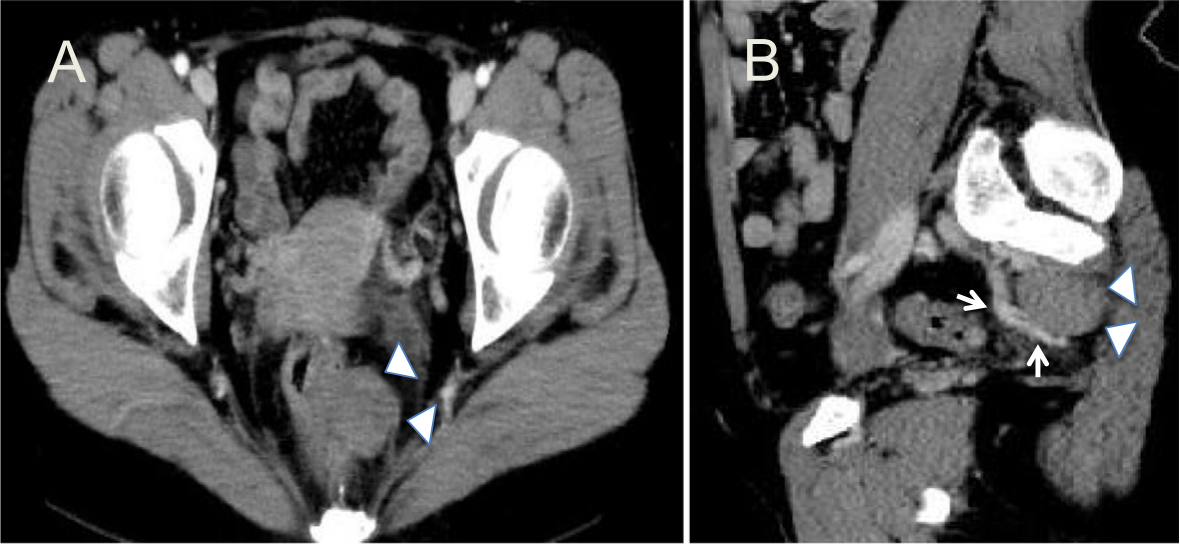
Contrast-enhanced computed tomography. **a** The multilocular cystic lesion has a diameter of approximately 32 mm, and no enhancing solid components or calcifications are visible (white arrowhead). **b** The blood supply arises from the middle sacral artery (white arrow)

On contrast-enhanced pelvic computed tomography (CT), the multilocular cystic lesion had a diameter of approximately 32 mm and was without enhanced solid components or calcification (white arrowhead). Blood was supplied to the lesion from the middle sacral artery (white arrow). There were no signs of lymph node metastasis (Fig. 2).

**Fig. 3:**
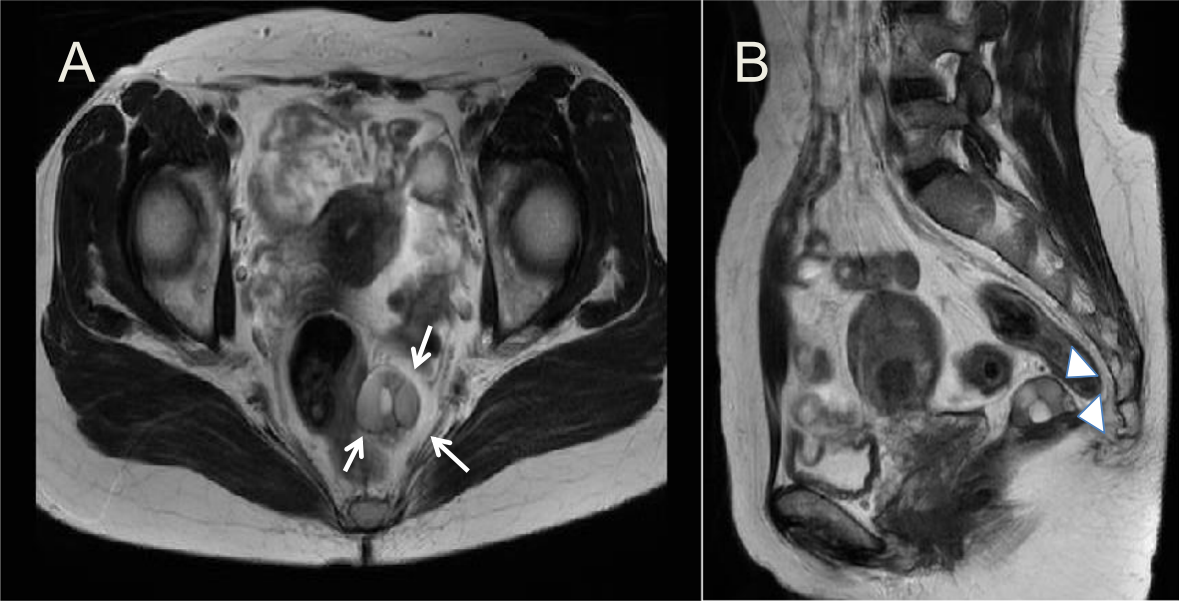
Magnetic resonance imaging. **a** On T2-weighted coronal magnetic resonance imaging, it appears that the 30-mm multicystic tumor on the left side of the posterior rectum is strongly adhered to the rectal wall (white arrow). **b** The enhancing cystic portions (white area in the center of the tumor) are accompanied by solid components (gray area at the margin of the tumor) (white arrowhead)

The finding of contrast enhancement of the cyst wall on magnetic resonance imaging (MRI) led us to suspect that the mass was strongly adhered to the left rectal wall (white arrows). The MRI also showed non-enhancing cystic components accompanied by solid components (white arrowhead) (Fig. 3).

The presumptive diagnosis was retrorectal tailgut cyst, and we decided to perform a laparoscopic tumor resection because we could not exclude the possibility of malignancy.

**Fig. 4:**
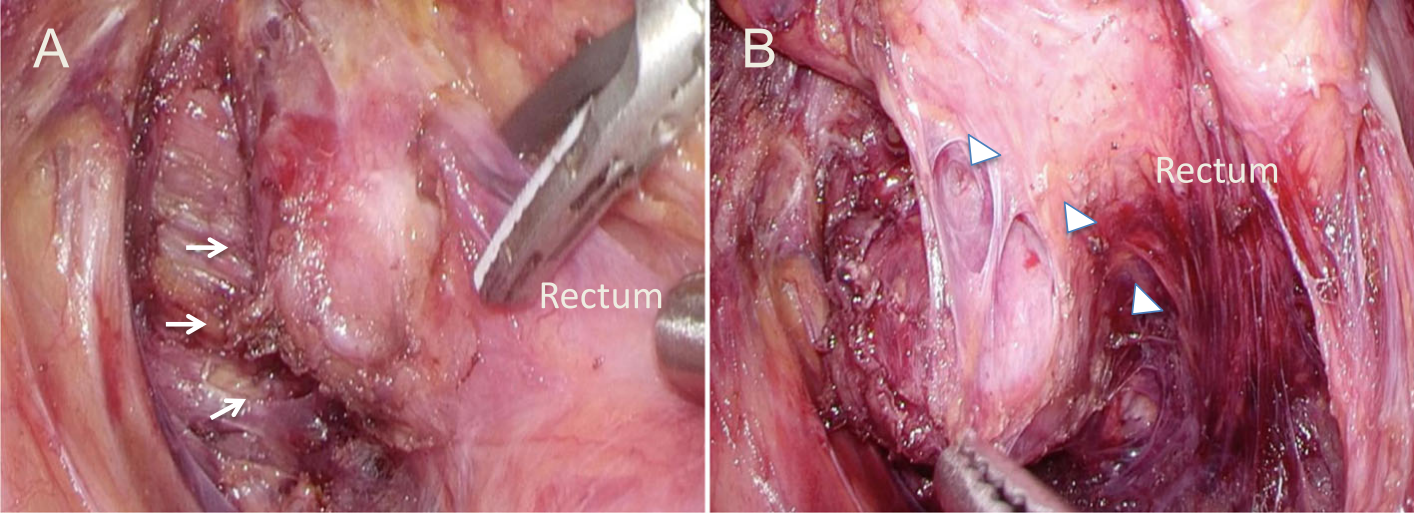
Intraoperative photos. **a** En-bloc resection of the tumor from the pelvic muscle fascia (white arrows). **b** The white arrowheads show the cut surface of the upper edge of the rectum. The tumor had infiltrated into the left posterior wall of the rectum

At laparoscopy, there was no evidence of liver metastasis or peritoneal dissemination. The capsule wall was free along the fascia propria of the rectum and could be seen in the left posterior wall of the rectum, free along the dorsal side of the tumor to the caudal side of the tumor (Fig. 4). The blood supply from the middle sacral vessels was divided and ligated with clips. An area of sclerosis along the tumor at the lateral rectum, which appeared to be the wall of the capsule, was breached during the resection, and a small amount of intestinal content was leaked. The pelvic cavity was copiously irrigated to prevent contamination. The tumor resection boundary was revised to completely remove the tumor, and the posterior and left side walls of the rectum were then reapproximated and repaired with eight stitches of 3–0 absorbable suture. Repeated checks for leaks were negative, but the digital rectal examination revealed a weakness in the rectal wall, which was secured by two additional stitches. The total interoperative blood loss was 270 mL, and the operation time was 368 min. The patient’s postoperative recovery was uneventful, and she was discharged without incident on postoperative day 7. Defecation dysfunction persisted for 2 months after surgery, but it subsequently resolved spontaneously.

**Fig. 5:**
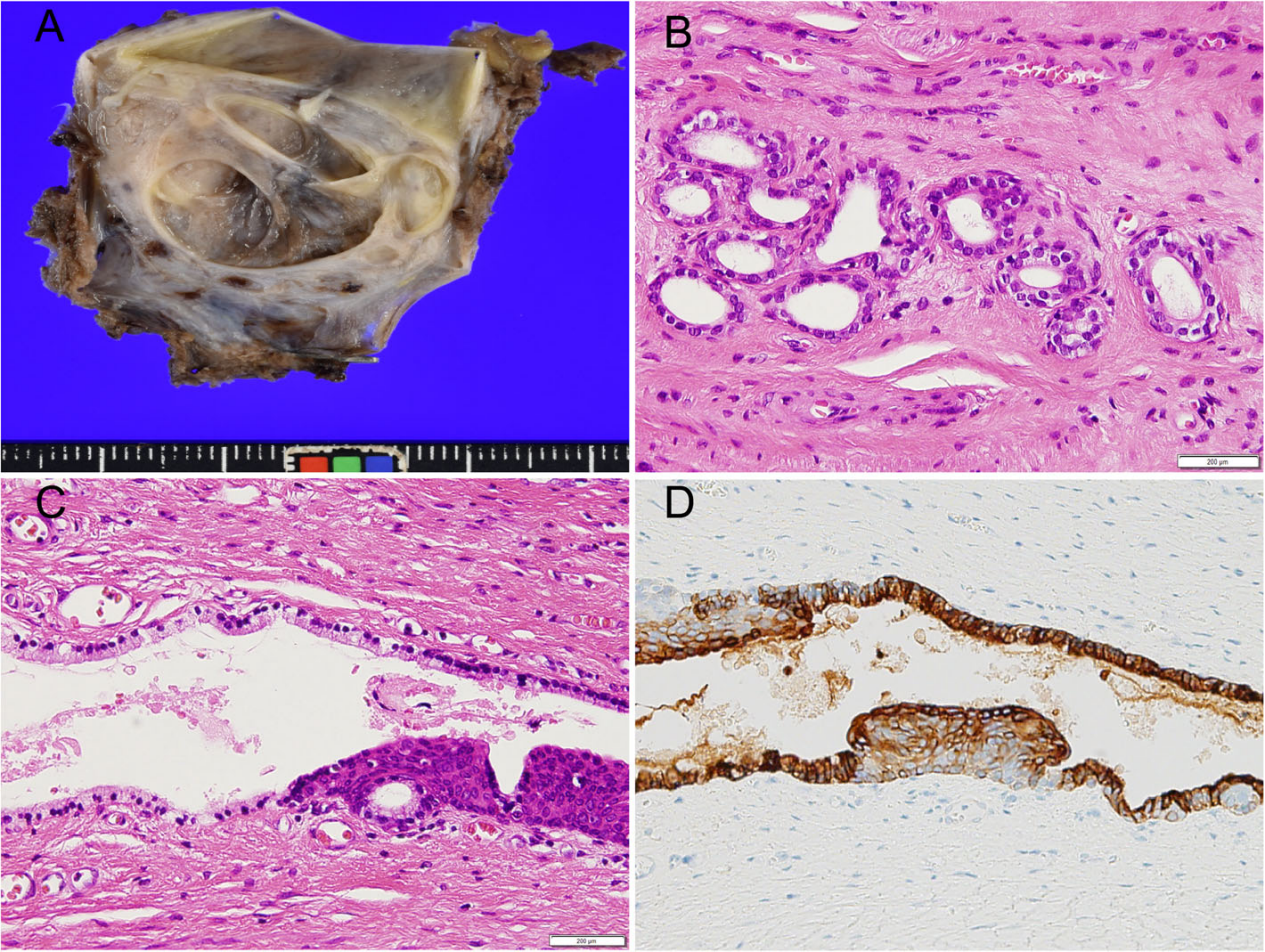
Histopathology. **a** Macroscopically, the tumor measures 43 × 35 × 10 mm and contains multiple cysts of different sizes. **b** The inner surfaces of the cysts are smooth and even (hematoxylin and eosin [HE] × 100). **c** The cyst walls are lined by eccrine sweat glands (HE × 200). **d** Immunostaining shows S100-positive myoepithelial cell-like cells (× 100)

Macroscopically, the tumor measured 43 × 35 × 10 mm and contained multiple cysts of different sizes, which contained white mucinous contents. Histologically, the inner surfaces of the cysts were smooth and even and the cyst walls were composed of smooth muscle, glial fibers, nerves, blood vessels, and fat. The immunohistochemical examination showed that the lesion had biphasic properties, containing tubular sweat gland-like structures composed of secretory cell-like cells as well as S100-positive myoepithelial cell-like cells, suggesting that the lesion contained eccrine sweat glands (Fig. 5). Cytokeratin (CK) 7, CK20, C-KIT, and adipophilin immunostaining were all negative, and the final diagnosis was a mature teratoma.

## Discussion

Retrorectal tumors are extremely rare, with a reported occurrence of 1 in 40,000 registered patients [7]. Developmental cysts of the retrorectum include tailgut cysts, epidermoid cysts, dermoid cysts, teratomas, and rectal intussusception [8]. Retrorectal teratomas in adults are mostly asymptomatic, and they are usually detected incidentally during clinical examinations or imaging studies [9].

Preoperative imaging including CT, MRI, and CE-US can assist with proper preoperative diagnosis of retrorectal tumors. CT can provide information about the size and density of the tumor and its spatial relationships, but it lacks specificity for differentiating between tumor types [10]. MRI is useful for determining the extent of retrorectal tumors and their relationships with surrounding structures [11].

Recently, CE-US has also been utilized for clinical diagnosis. However, it is still difficult to distinguish a teratoma from a tailgut cyst by this method, as was demonstrated in our case. Preoperative biopsy of retrorectal tumors is generally not recommended because of the risk of infection or tumor seeding in the pelvis. As such, definitive diagnosis is best obtained following complete resection of the tumor [7]. Resection of retrorectal teratoma is generally regarded as appropriate because of the malignant potential [2, 3, 12, 13]. If malignant transformation has occurred, only microscopic radical resection with clear resection margins can provide good outcomes [14].

The first laparoscopic removal of a benign pelvic retroperitoneal cyst was reported in 1995 [15], and there have been sporadic reports about similar laparoscopic procedures in recent years. The main reported advantages of laparoscopic surgery in such cases include smaller wounds, less postoperative pain, a good field of vision, and the ability to accurately discriminate between the tumor and adjacent structures [16, 17]. In the current case, we successfully performed laparoscopic tumor resection in spite of the tumor infiltration in the rectal wall. Laparoscopy provides a good field of vision in the narrow space behind the rectum, and the magnification effect permits the surgeon to see the detailed anatomy of the pelvic floor more clearly. In addition, our case demonstrated that the narrow pelvic laparoscope was effective in suturing the injured posterior wall of the rectum, reducing trauma. A drawback of the laparoscopic approach is the lack of direct touch, which may make it difficult to precisely discern the tumor boundaries.

## Conclusion

In summary, we describe a very rare case of a mature retrorectal teratoma. The laparoscopic management of mature retrorectal teratomas is feasible and promising because of its reduced invasiveness and the fact that it can be adapted without extending the skin incision.

## Data Availability

The datasets supporting the conclusions of this article are included within the article and its additional files.

## Abbreviations

CE-US: Contrast-enhanced ultrasonography
CT: Computed tomography
EUS: Endoscopic ultrasonography
HE: Hematoxylin and eosin
IHC: Immunohistochemical
US: Ultrasonography
